# Isochromosome Mosaic Turner Syndrome With Epilepsy and Developmental Abnormalities: A Case Report

**DOI:** 10.7759/cureus.50961

**Published:** 2023-12-22

**Authors:** Cesar Prugue, Lindsay Tjiattas-Saleski, Steven Enkemann

**Affiliations:** 1 Medical School, Edward Via College of Osteopathic Medicine-Carolinas, Spartanburg, USA; 2 Emergency Medicine, Edward Via College of Osteopathic Medicine-Carolinas, Spartanburg, USA; 3 Genetics, Edward Via College of Osteopathic Medicine-Carolinas, Spartanburg, USA

**Keywords:** anti-epileptic drugs, developmental abnormalities, isochromosome mosaic turner syndrome, epilepsy, turner syndrome

## Abstract

Turner syndrome (TS) is a genetic disorder resulting from the partial or complete absence of one X chromosome in females. This condition gives rise to a spectrum of physical and developmental characteristics. Isochromosome mosaic Turner syndrome (IMTS) is a distinct form of this disorder, characterized by genetically different cell lineages, where one or more of the lineages contain an isochromosome X consisting of either p or q arms. While IMTS is relatively common, the relationship between IMTS and epilepsy along with developmental abnormalities remains an area of further investigation.

An eight-year-old female presented with a seizure lasting more than 10 minutes, subsequent bilateral hand weakness, and an abnormal gait. Neurologic evaluation revealed a 24-hour amplitude-integrated electroencephalogram (EEG) demonstrating generalized sharp and slow waves increased with hyperventilation and left-sided delta activity. Both the brain magnetic resonance imaging (MRI) and repeated EEG, conducted while the patient was sedated, showed normal results. The patient was diagnosed with generalized epilepsy with underlying left hemispheric dysfunction. Early medical history revealed acid reflux, heightened sensitivity or aversion to certain textures, swallowing difficulties, attention-deficit/hyperactivity disorder, extremity clumsiness, and a focal seizure one year prior. In the following months, the patient continued having generalized tonic-clonic seizures and developed bilateral muscle weakness in her arms and legs. This prompted genetic testing, which revealed a karyotype of 45,X,t(17;20)(q23;p13)/46,X,I(X)(q10),t(17;20)(q23;p13) consistent with IMTS and an additional chromosomal translocation.

This rare case highlights the potential association between IMTS and the development of epilepsy, emphasizing the importance of a multidisciplinary approach in evaluating TS patients. There is a need for further research that explores the genetic link between TS variants and epilepsy, as well as other intellectual disorders.

## Introduction

Turner syndrome (TS) is characterized by a complete or partial loss of one X chromosome in females, with an incidence rate of 1 in 2000 to 1 in 3000 live female births [1]. This congenital condition has been associated with decreased survival rates compared to age-matched controls, with cardiovascular disease identified as the major factor [2]. Various TS karyotypes have been reported, with 45,X and mosaic TS variants like 45,X/46,X iso (Xq) being the most common [3]. TS presents a diverse range of clinical manifestations including short stature, cardiac anomalies, renal abnormalities, audiologic abnormalities, and reproductive dysfunction [4]. Additionally, TS has been associated with an increased risk of neurocognitive and psychosocial differences [5]. While epilepsy is not common in TS, it has been reported in a limited number of case reports, each with varying presentations and karyotypes [6]. Isochromosome mosaic Turner syndrome (IMTS) is a variant TS characterized by the presence of cells that have karyotypes differing from the typical 45,X arrangement, accompanied by anomalies in the structure of the X chromosome arms. IMTS has a prevalence of 8-9% in females with TS [7]. The karyotype of this patient indicates a variant IMTS due to the presence of a chromosomal translocation. The nature of this translocation, whether de novo or hereditary, remains unknown since genetic testing has not been conducted on the parents. Importantly, it is worth noting that even in the absence of a chromosomal translocation, IMTS with epilepsy has not been identified in the existing literature.

This report will provide a detailed description of a patient with a unique TS karyotype who presented with epilepsy, developmental delay, mild hypotonia, and intellectual disability. An overview of her clinical course and management strategies will be described.

## Case presentation

**Figure 1:**
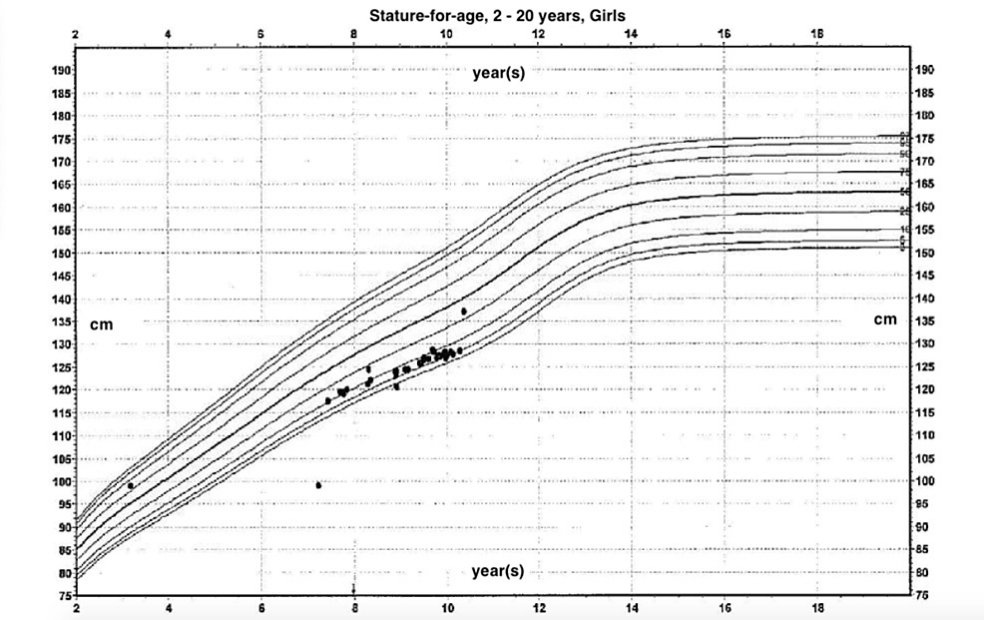
The patient’s growth chart

The patient was born at 36 weeks via cesarean section, weighing eight pounds and three ounces. The mother, a 31-year-old G4 P2012, experienced complications during pregnancy, including preeclampsia and maternal liver failure. She was taking flupentixol (Depixol®) during pregnancy due to previous postpartum depression. Family history revealed several conditions, including migraine, depression, attention-deficit/hyperactivity disorder (ADHD), bipolar disorder, and epilepsy in one grandparent. The patient’s two older sisters had no significant medical history. During infancy and childhood, the patient experienced acid reflux, food aversions, texture sensitivities, poor swallowing, mild developmental delay, ADHD, extremity clumsiness, and a focal seizure at age seven. This seizure occurred two weeks after beginning Focalin® XR for ADHD, after which the medication was discontinued. Her developmental milestones were consistently borderline during pediatric wellness visits (Figure 1), and at age seven she was evaluated to be borderline for cognitive gross motor and fine motor skills.

**Figure 2:**
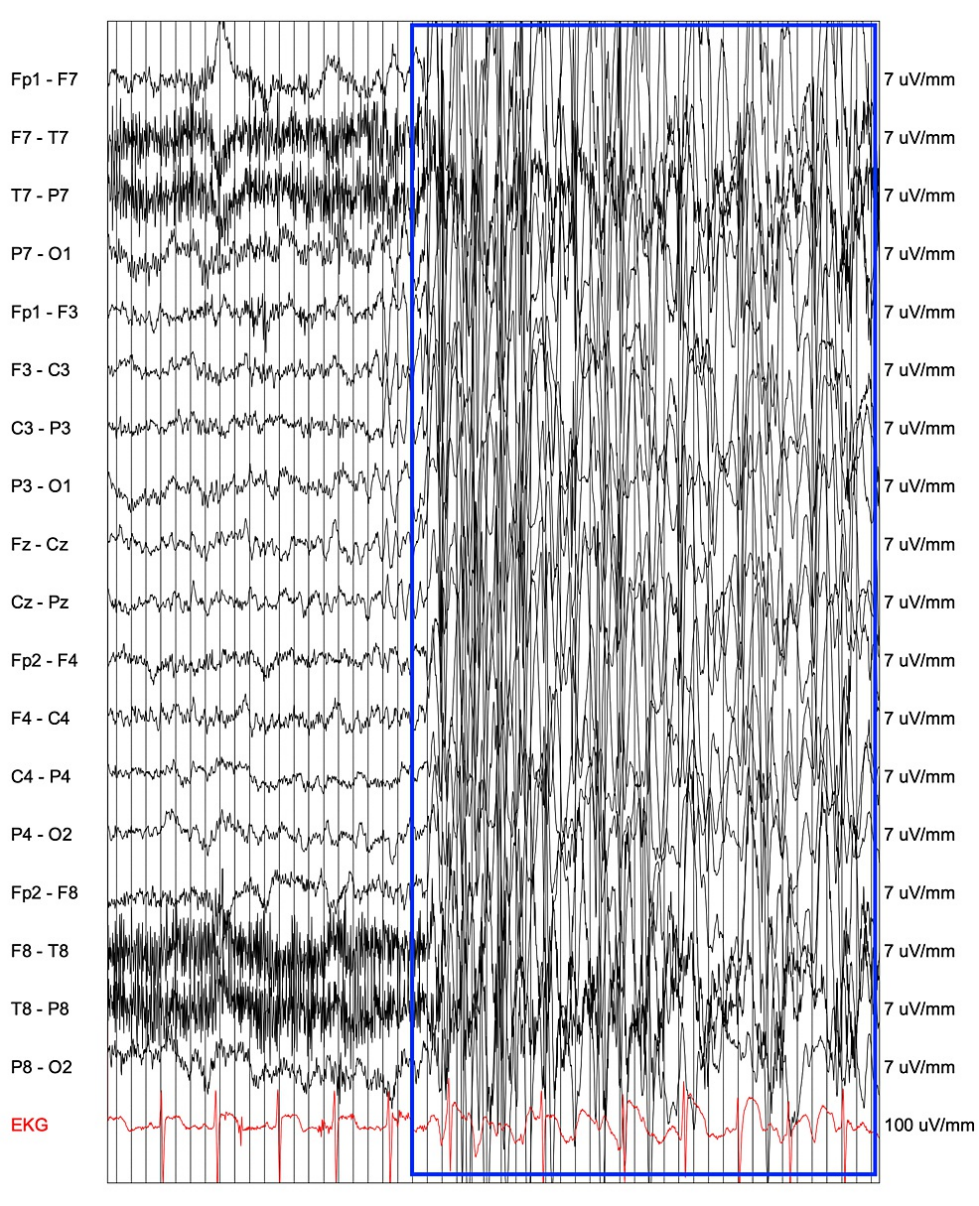
EEG reveals an abnormal pattern characterized by generalized epileptiform discharges, specifically bursts of 3-4 Hz spike and wave activity. This lasted for 50 seconds without any behavioral change.

**Figure 3:**
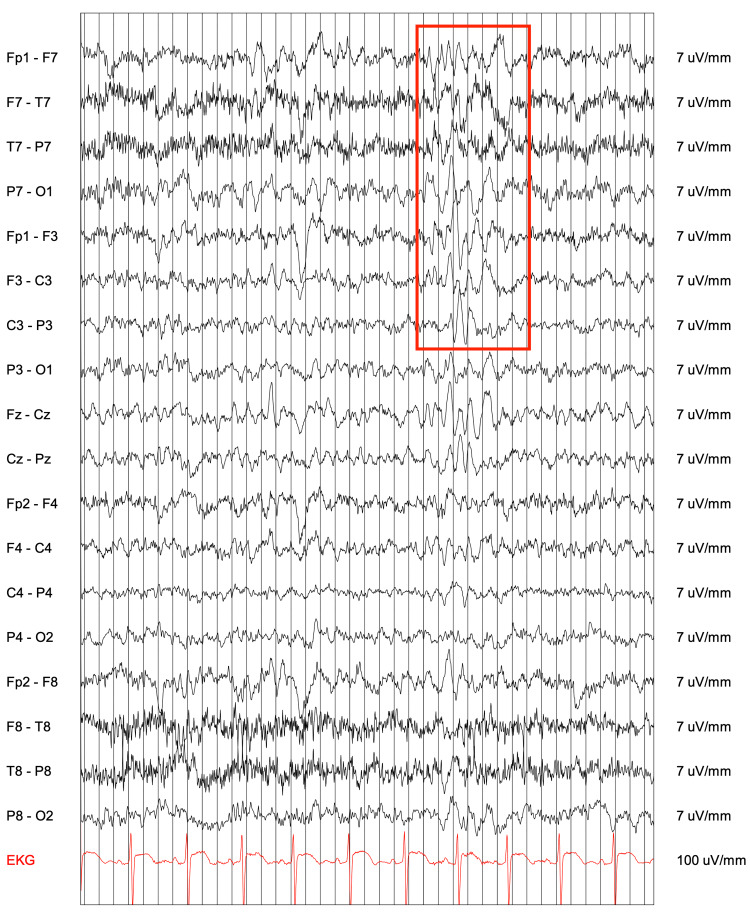
EEG shows left hemispheric slowing.

At age eight, the patient presented to the emergency room with a generalized tonic-clonic seizure. A 24-hour amplitude-integrated EEG showed generalized sharp and slow waves (Figure 2) increased with hyperventilation, and left-sided delta activity (Figure 3), suggesting epileptic activity. Brain MRI and EEG while sedated were normal. She was diagnosed with epilepsy featuring both generalized and focal features. Initial treatment with levetiracetam (100mg/mL 6 ml twice daily and progressively raised to 9 ml) was ineffective and she experienced more than 10 additional generalized tonic-clonic seizures in the following months. Subsequent treatment with lamotrigine (LTG, 25 mg progressively raised to 150 mg twice daily) resulted in a decrease in tonic-clonic seizures, but occasional focal seizures continued. After the onset of the seizure disorder, the patient developed persistent bilateral hand weakness and an abnormal gait involving the in-toeing of the left foot.

Due to the patient's seizure disorder, weakness, and fatigue, whole exome sequencing and mitochondrial sequencing were conducted at age nine. Results indicated a TS variant with two different cell lines, one with 45,X and the other with one normal X and one isochromosome for the long arm of X. No single gene disorders were detected. The mitogenome sequencing was negative. Karyotype testing followed and confirmed the exome results and revealed an unexpected balanced translocation between chromosomes 17 and 20 at breakpoints q23 and p13. The karyotype was 45,X,t(17;20)(q23;p13)/46,X,I(X)(q10),t(17;20)(q23;p13). The patient's parents were advised to undergo genetic testing to determine if the translocation was inherited or de novo, but they decided to not undergo testing as they were not planning additional children.

Physical examination findings at age 10 showed few TS features, including slightly down slanting palpebral fissures, a small and narrow palate with dental crowding, large but normal ears, and no webbed neck. Tanner stage 2 pubic hair and Tanner stage 1 breasts were noted. A bone age study predicted a final adult height of 5’1’’ (the mother’s height is 5’2’’). Growth hormone therapy was started (somatropin 2.0mg/day) but was only administered for a few years before being discontinued at the patient’s request. Estradiol patches were initiated at age 12 for hormone replacement therapy due to ovarian failure confirmed by high serum follicle-stimulated hormone (56.4 mIU/mL) and low estradiol levels (<5.0 pg/mL). As a result of a small focal seizure after starting estradiol, LTG was increased to 200 mg twice a day. One year later, the patient experienced a grand mal seizure, the first one in two and a half years. To control the convulsions, zonisamide 100 mg was added, while LTG was reduced to 175 mg due to dizziness side effects. The convulsions have been well controlled since these medication changes were initiated.

The educational delay was noted by the parents soon after the first tonic-clonic seizure at age eight, prompting her parents to pursue comprehensive neuropsychological evaluations to gain a better understanding of her conditions and needs. A thorough evaluation by a pediatric neuropsychologist indicated weaknesses in attention/working memory, processing speed, and visual-motor skills. It was concluded that the patient exhibited features associated with a non-verbal learning disorder (NVLD) and that her cognitive development was below average for her age. A follow-up evaluation at age 13 showed a slight decline in verbal intelligence and visual memory, which may be attributed to her recurrent seizure activity. In addition to the medications mentioned, the patient also takes atomoxetine 25 mg daily for inattention/anxiety and levothyroxine 50 mcg daily for hypothyroidism. From a cardiovascular perspective, she has a normal electrocardiogram and echocardiogram, which were checked as part of a routine evaluation to rule out any potential cardiac abnormalities associated with TS.

## Discussion

The intersection of TS and epilepsy is a compelling area of investigation within the medical field. While TS is reported to occur in about 1 in 2500 live female births, the true prevalence remains unknown due to undiagnosed individuals with milder phenotypes [8]. In contrast, epilepsy is a prevalent neurological disorder affecting roughly 1% of the general population [9]. The occurrence of epilepsy as a comorbidity in TS is not common, presenting in about 3% of cases [10]. These statistics emphasize the unique nature of epilepsy in TS patients, necessitating individualized treatment approaches to address the coexisting clinical features.

While the association between TS and epilepsy has been documented in several case reports, the coexistence of these conditions is relatively infrequent. Akasaka et al. reported a case of a patient with mosaic TS who experienced intractable epilepsy [6]. Similar to the patient in this report, their case involved generalized tonic epilepsy presenting in childhood. A variety of anti-epileptic drugs (AEDs) were attempted, with a combination therapy of LTG and valproic acid (VPA) proving effective in significantly reducing seizure frequency. LTG and VPA were used in the published case because this combination therapy showed positive outcomes in previous reviews. Balancing the need for effective antiepileptic action with long-term side effects can be particularly challenging when these two conditions co-exist.

In the present case, LTG was utilized as monotherapy for a few years until zonisamide was introduced as an adjunctive treatment. It's worth noting that VPA is commonly used as monotherapy due to its higher efficacy as compared to LTG, but it's important to consider long-term adverse effects associated with VPA, particularly on cognitive function. Studies have shown that LTG can have a positive effect on pediatric epilepsy patients with ADHD symptoms [11], which aligns with the ADHD history observed in the patient. Furthermore, zonisamide as an adjunct therapy has demonstrated effectiveness and good tolerability in pediatric patients [12]. Additionally, drug interactions should be taken into account when managing these coexisting conditions. Patients with TS may require hormone replacement therapy, including estrogens, which are metabolized by CYP3A4. Many antiepileptic drugs are known CYP3A4 inducers, potentially affecting the effectiveness of hormone replacement therapy and growth hormone replacement [13]. This underscores the importance of individualized treatment approaches. In our unique case, the patient received a symptomatic approach, carefully considering drug interactions. The absence of a predefined intervention protocol highlights the need for further research to explore the potential benefits and challenges of specific drug combinations in such cases.

This patient's karyotype analysis revealed a variant isochromosome mosaic TS (IMTS) with the following karyotype: 45,X,t(17;20)(q23;p13)/46,X,I(X)(q10),t(17;20)(q23;p13). Similarities to this case were observed in other IMTS reports, where patients exhibited mild phenotypic characteristics typical of TS [7,14]. Understanding whether the epilepsy in this patient has a genetic or hereditary basis is challenging due to the complex nature of epilepsy genetics. While specific genes like cyclin-dependent kinase-like 5 (CDKL5), neurite extension and migration factor (NEXMIF), aristaless related homeobox (ARX), and methyl-CpG binding protein 2 (MECP2), which are linked to seizures, should be considered, epilepsy is not always directly linked to a single gene. Research suggests the X chromosome's importance in brain development and function [15], but further investigation is needed to identify any specific genetic factors. Additionally, this patient's mosaic karyotype and X-inactivation further complicate the gene expression patterns, with different cells expressing either the intact X, the isochromosome, or both, depending on the translocation. Epilepsy and intellectual disorders may be more common in TS than the literature suggests, highlighting the complexity of caring for TS patients and the limited availability of researched treatment regimens for such complex cases [16]. Early genetic testing is critical for providing prompt diagnosis and treatment in suspected TS cases [17].

## Conclusions

This is a unique case presentation of an individual with IMTS, epilepsy, and developmental abnormalities. Effective epilepsy management required exploring diverse AEDs at varying dosages, eventually achieving control through a combination therapy of LTG and zonisamide. This highlights the intricate care needed for TS patients and the importance of a multidisciplinary specialist team. Continued collaboration between healthcare professionals and researchers is essential to offer optimal care for TS patients with epilepsy.
